# Transvection-like interchromosomal interaction is not observed at the transcriptional level when tested in the *Rosa26* locus in mouse

**DOI:** 10.1371/journal.pone.0203099

**Published:** 2019-02-14

**Authors:** Keiji Tanimoto, Hitomi Matsuzaki, Eiichi Okamura, Aki Ushiki, Akiyoshi Fukamizu, James Douglas Engel

**Affiliations:** 1 Faculty of Life and Environmental Sciences, Life Science Center for Survival Dynamics, Tsukuba Advanced Research Alliance (TARA), University of Tsukuba, Tsukuba, Ibaraki, Japan; 2 Graduate School of Life and Environmental Sciences, University of Tsukuba, Tsukuba, Ibaraki, Japan; 3 Graduate School of Biomedical Sciences, Tokushima University, Tokushima, Japan; 4 Department of Cell and Developmental Biology, University of Michigan, Ann Arbor, United States of America; Hirosaki University Graduate School of Medicine, JAPAN

## Abstract

Long-range associations between enhancers and their target gene promoters have been shown to play critical roles in executing genome function. Recent variations of chromosome capture technology have revealed a comprehensive view of intra- and interchromosomal contacts between specific genomic sites. The locus control region of the β-globin genes (β-LCR) is a super-enhancer that is capable of activating all of the β-like globin genes within the locus in *cis* through physical interaction by forming DNA loops. CTCF helps to mediate loop formation between LCR-HS5 and 3’HS1 in the human β-globin locus, in this way thought to contribute to the formation of a “chromatin hub”. The β-globin locus is also in close physical proximity to other erythrocyte-specific genes located long distances away on the same chromosome. In this case, erythrocyte-specific genes gather together at a shared “transcription factory” for co-transcription. Theoretically, enhancers could also activate target gene promoters at the identical loci, yet on different chromosomes *in trans*, a phenomenon originally described as transvection in *Drosophilla*. Although close physical proximity has been reported for the β-LCR and the β-like globin genes when integrated at the mouse homologous loci in *trans*, their structural and functional interactions were found to be rare, possibly because of a lack of suitable regulatory elements that might facilitate such *trans* interactions. Therefore, we re-evaluated presumptive transvection-like enhancer-promoter communication by introducing CTCF binding sites and erythrocyte-specific transcription units into both LCR-enhancer and β-promoter alleles, each inserted into the mouse *ROSA26* locus on separate chromosomes. Following cross-mating of mice to place the two mutant loci at the identical chromosomal position and into active chromation in *trans*, their transcriptional output was evaluated. The results demonstrate that there was no significant functional association between the LCR and the β-globin gene *in trans* even in this idealized experimental context.

## Introduction

Gene expression is tightly regulated by DNA *cis* elements and their binding *trans*-factors, in which specific enhancer-promoter communications play a pivotal role. While genome-wide sequencing of the human and mouse genomes disclosed the number of genes to be more than 20,000, that of enhancer elements is predicted to far exceed the number of genes [1]. Because accumulating evidence suggests that perturbation of enhancer function can be a major cause of pathogenesis in human diseases [2], it is of paramount importance to assign the activity of any individual enhancer to a specific target gene(s) in order to predict its function. Genome-wide interactome analyses revealed that enhancers can *physically* interact with genes over enormous distances, exceeding several hundreds of kilobase pairs in *cis* [3], or even with genes located on different chromosomes in *trans* [4], indicating the presence of molecular mechanisms that allow specific enhancer-promoter interactions to take place over very long distances.

In the interphase nucleus, the genome adopts a higher-order chromatin architecture, in which transcription factors play important roles. Among those, CTCF, first identified as a transcriptional activator or repressor and subsequently, as an insulator, binds to two distinct genome regions to bring those two sites into close spatial proximity [5–7]. Ineractome analysis by ChIA-PET in ES cells revealed that the number of intra- or interchromosomal interactions mediated by CTCF was 1,480 and 336, respectively [8]. More sensitive HiChIP experiments in the human B lymphocyte cell line identified in the order of 10,000 cohesin (a functional partner of CTCF)-mediated interactions [9]. However, how frequently gene expression is reflected by changes in CTCF-mediated genome architecture is not well understood. On the other hand, it has been reported that genes with similar transcriptional specificity migrate into transcription factories in the nucleus that are rich in transcription factors engaged in the expression of those genes [10–12]. According to this mechanism, two distinct genome regions carrying genes with the same expression pattern should meet at the shared foci for co-transcription.

**Fig 1:**
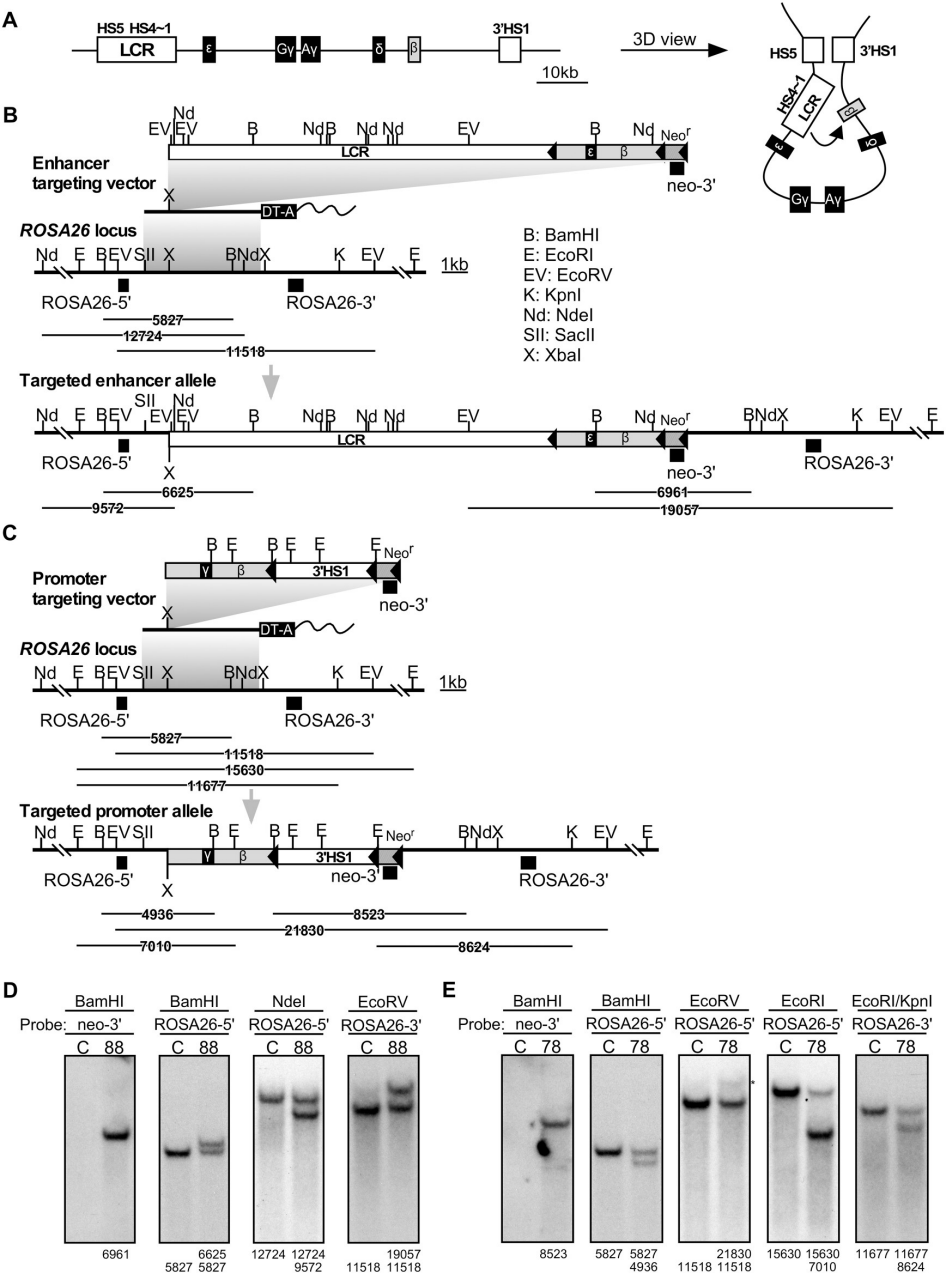
Generation of enhancer and promoter knock-in alleles in mice. **(A)** Structure of the human β-globin gene locus shown in 1D (left) and 3D (right) views. **(B)** The enhancer targeting vector carrying the human β-globin LCR and β-globin gene that is marked by an ε-globin sequence, wild-type *ROSA26* locus, and the correctly targeted enhancer knock-in locus are shown. In the targeting vector, neomycin resistance (Neo^r^) and diphtheria toxin (DT)-A genes are shown as striped and solid boxes, respectively. The solid triangles indicate the loxP sequences. Probes used for Southern blot analyses in (D) are shown as filled rectangles. Expected restriction fragments with their sizes are shown beneath the partial restriction enzyme maps. **(C)** The promoter targeting vector carrying the human β-globin gene (marked by an γ-globin sequence) and 3’HS1, wild-type *ROSA26* locus, and the correctly targeted promoter knock-in locus are shown. Probes used for Southern blot analyses in (E) are shown as filled rectangles. **(D and E)** Genomic DNA from ES clones was digested with restriction enzymes, separated on agarose gels, and Southern blots were hybridized to the probes. Asterisks denote nonspecific bands.

The human β-like globin genes are organized within a 70-kbp span on human chromosome 11, with the embryonic ε-globin gene located most 5′, followed by the two fetal γ-globin genes (Gγ and Aγ), while the adult δ- and β-globin genes are at the 3′ end of the locus (Fig 1A). Expression of all the β-like globin genes in primitive, as well as in definitive erythroid cells, depends on the activity of the locus control region (LCR; [13, 14]), a super-enhancer element located 48 kbp 5′ to the transcription initiation site of the β-globin gene. The LCR consists of five DNaseI hypersensitive sites (HSSs), among which HS1 to 4 are constituent enhancers and rich in binding sites for transcription factors [15–17], while HS5 carries CTCF binding sites [18].

How the distal LCR enhancer activates β-globin gene expression has long been a subject of intense debate [19]. In 2002, RNA TRAP [20] and chromatin conformation capture (3-C; [21, 22]) assays elegantly revealed that the LCR and β-globin promoters were positioned in close proximity: these observations were consistent with a looping model, in which proteins bound to the LCR enhancer and to the gene promoters physically interact with the intervening DNA sequences looped out [23].

Erythroid specific transcription factors, such as GATA-1, NF-E2 and EKLF are essential for efficient globin genes transcription through binding to both the LCR and globin gene promoters. It is therefore presumed that they participate somehow in long-range enhancer-promoter interactions. In fact, both GATA-1 and NF-E2 are essential for LCR and β-maj-globin proximity in murine erythroid cells [24, 25], as well as for LCR and γ-globin proximity in human erythroid cells [26]. Similarly, EKLF is also required for loop formation between the LCR and β-globin promoter sites [27]). Because non-genic LCR sequences are transcribed in erythroid cells [28, 29], LCR and β-globin gene may be co-transcribed in the same RNA polymerase II (PolII) factory, which then aids their physical association and transcriptional activation of the β-globin gene by the LCR enhancer. In accord with this notion, the β-globin gene locus on mouse chromosome 7 was found to colocalize with erythroid specific genes located 20 Mb away on the same chromosome in erythroid cell nuclei [10]. Furthermore, the murine β-globin gene locus colocalized with the *Slc4a1* (chromosome 11) and *Cd47* (chromosome 16) genes at a shared polII factory [12].

Interestingly, CTCF binding was found around the HSSs at both ends of the locus, *i*.*e*. LCR-HS5 and the 3’HS1 regions (Fig 1A; [21]). Although 3C assays revealed proximal positioning of these sites in the nucleus, it was not confined to erythroid cells. In globin expressing cells, the LCR and 3’HS1 regions are further located proximally to the actively expressed β-globin genes, which structure has been termed an active chromatin hub (Fig 1A; [21, 30]). Therefore, transcriptional activation of the β-like globin genes is predicted to be a multi-step process, in which HS5-3’HS1 interaction may help to bring LCR enhancer sequences within close proximity of the β-globin promoter, thus facilitating their productive interaction (Fig 1A).

Because the LCR makes direct contact with its targets through looping mechanism, it can theoretically touch and activate such targets on separate chromosomes. Such interchromosomal interaction for functional enhancer-promoter communication was dubbed “transvection” in *Drosophilla* [31, 32]. In the case of the *yellow* locus, for example, an enhancer of one copy of a gene (that lacks promoter activity) regulates the expression of the paired copy of the gene (lacking enhancer activity) in *trans*. As mentioned earlier, 3C-based biochemical strategies have identified several examples of *functional* interchromosomal interactions also in the mammalian genome [33–36]. In the latter “trans-interaction” cases, however, precise pairing of the two homologous alleles seemed not mandatory for enhancer-promoter communication.

To test whether interchromosomal *functional* as well as physical association between the LCR enhancer and the β-like globin gene promoters take place, Noordermeer *et al*. knocked-in each sequence at a gene-dense site in the mouse genome separately on homologous chromosomes [37, 38]. Because they found upregulation of endogenous murine β-like globin genes (βh1; ~2-fold) on the separate chromosome, they concluded that the LCR must have some affinity for the β-globin promoter even in *trans*. However, neither interchromosomal homologous chromosome interactions nor transvection-like activation of reporter genes was observed.

Although CTCF binding to the HS5 in the ectopic LCR was observed, CTCF-assisted or co-transcriptionally mediated mechanisms seemed not fully considered in their experimental design [37]. We therefore decided to re-evaluate transvection in mammals by incorporating well-characterized β-globin *cis* elements at the *ROSA26* locus. Firstly, LCR-HS5 and 3’HS1 sequences were introduced into enhancer and promoter alleles, respectively, in expectation that CTCF factors bound at these sites might promote the formation of an interchromosomal bridge, which would in turn facilitate functional interactions between the LCR enhancer elements (HS4~1) and the β-globin promoter. In addition, β-globin transcription units were included in both alleles, anticipating that two alleles bearing the same promoter would likely migrate into same PolII factory in the nucleus. Even in this idealized experimental design, however, transvection-like functional association between two alleles on separate chromosomes was not observed.

## Materials and methods

### Targeting vectors

Floxed neomycin resistance gene (flNeo^r^) cassete was released from pMC1neopA_5'/3'-loxP [39] by *Xba*I/*Nhe*I digestion and inserted into *Xba*I site of pROSA26-1 (generous gift of Dr. Philippe Soriano; nucleotide position at 180,029 in AC155722; RP24-204J8) to generate pROSA26/MC1neopA_5'/3'-loxP(-).

5'-upstream portion of human β-globin gene (nucleotides 60,577–60,882 in HUMHBB; U01317.1; GenBank) was PCR-amplified by using a set of oligonucleotides: ICI-02-5S; 5'-GGGGTACC TCTAGATCTCTATTTATTTAGCA-3' (artificial *Kpn*I and *Xba*I, and endogenous *Bgl*II [at 60,557] sites are underlined) and ICI-02-3A; 5'-GGTCAGCGTAGGGTCTCAGT-3'. Following *Kpn*I and *Apa*I (at 60,882) digestion, this fragment and 3'-downstream portion of the gene (*Apa*I-*Xba*I fragment; nucleotides 60,882–65,426) were linked and cloned into *Kpn*I/*Xba*I sites of pBluescriptII KS(+). *Bam*HI site (at 60,676) of this plasmid was then eliminated to generate pβ-globin_K-X-ΔB for facilitating cloning procedure.

Portions of ε- and Aγ-globin gene sequences were PCR-amplified by using following two sets of oligonucleotides: ICI-04-5S; 5'-GGCACCATGGTGCATTTTACTGCT-3' (artificial *Nco*I site underlined) and ICI-03-3A; 5'-TCAGGATCCACATGCAGCTT-3' (*Bam*HI) or ICI-03-5S; 5'-CACACACTCGCTTCTGGAAC-3' and ICI-03-3A, respectively. Following *Nco*I (partial) and *Bam*HI digestion, ε (nucleotides 19,539–19,959) and Aγ (nucleotides 39,465–39,885) gene sequences were replaced with corresponding portion of β-globin gene (nucleotides 62,185–62,613) in pβ-globin_K-X-ΔB to generate pmβ/ε and pmβ/γ, respectively.

[Promoter targeting vector]

Following two oligonucleotides were annealed, phosphorylated and inserted into *Nde*I site (at 65,287 in HUMHBB) of the β-globin gene in pmβ/γ to generate pmβ/γ_loxP(-): BTLX-5S; 5'-TATCGGATCCT*ATAACTTCGTATAATGTATGCTATACGAAGTTAT*AGA-3' and BTLX-3A; 5'-TATCT*ATAACTTCGTATAGCATACATTATACGAAGTTAT*AGGATCCGA-3'. In each oligo, loxP sequences are italisized and *Bam*HI sites underlined. The *Xba*I fragment, carrying β-globin promoter, γ sequence-marked β-globin coding region, and a loxP site, was released from pmβ/γ_loxP(-) and introduced into *Xba*I site of pROSA26/MC1neopA_5'/3'-loxP(-) to generate pR26/loxP-Neo/mβ/γ.

Human β-globin 3'HS1 sequence (4,194 bp, *Sma*I-*Hin*dIII) was subcloned from BAC clone, RP11-1205H24 (nucleotides 35,934–40,127 in AC129505; GenBank). Upon conversion of *Hin*dIII site to *Sma*I site, the fragment was introduced into *Sma*I site of pR26/loxP-Neo/mβ/γ to generate pR26/loxP-Neo/mβ/γ/3'HS1 (Promoter targeting vector).

[Enhancer targeting vector]

Following two oligonucleotides were annealed, phosphorylated and inserted into *Bgl*II site (at 60,557 in HUMHBB) of the β-globin gene in pmβ/ε to generate pmβ/ε_loxP(-): ICI-08-5S; 5'-GATCGGCGCGCC*ATAACTTCGTATAATGTATGCTATACGAAGTTAT*-3' and ICI-08-3A; 5'-GATC*ATAACTTCGTATAGCATACATTATACGAAGTTAT*GGCGCGCC-3'. In each oligo, loxP sequences are italisized and *Asc*I sites underlined. The *Xba*I fragment, carrying a loxP site, β-globin promoter, and ε sequence-marked β-globin coding region, was released from pmβ/ε_loxP(-) and introduced into *Xba*I site of pROSA26/MC1neopA_5'/3'-loxP(-) to generate pR26/loxP-Neo/mβ/ε.

For facilitating cloning procedure, following two oligonucleotides were annealed, phosphorylated and inserted into *Kpn*I/*Sac*I site of pBluescriptII KS(+): ICI-01-5S; 5'- GGCGCGCCGGTACCTATGCGGCCGCGGCGCGCCAGCT-3' and ICI-01-3A; 5'-GGCGCGCCGCGGCCGCATAGGTACCGGCGCGCCGTAC-3', which resulted in *Asc*I-*Kpn*I-*Not*I-[*Sac*II]*-Asc*I sites formation. Human β-globin LCR sequences (17,590 bp, *Eco*RI-*Xba*I) were recovered from pRS/LCR [40] as *Kpn*I-*Not*I (both in the multicloning sites) fragment and inserted into *Kpn*I-*Not*I site of above plasmid to generate pBS/LCR. Upon eliminating *Sac*II site (parenthesized site in the above double-stranded oligo) from pBS/LCR, 3' downstream region of HS1 was accidentally deleted and the final LCR size cloned was (17,198 bp, nucleotides 95,131–77,934 in AC104389; Ensemble). Finally, LCR sequences were recovered from pBS/LCR as *Asc*I fragment and introduced into *Asc*I site of pR26/loxP-Neo/mβ/ε to generate pR26/LCR/loxP-Neo/mβ/ε (Enhancer targeting vector).

### Gene targeting in ES cells and generation of mutant mice

Target vectors were linearized by *Sac*II digestion. R1-ES cells were grown on embryonic fibroblast feeder cells. Following electroporation (Bio-Rad GenePulser Xcell [0.4 mm gap] at setting of 250 V and 500 microfarads) of cells (1.0 x 10^7^ cells) with a linearized targeting vector (20 μg), cells were selected in 0.4 mg/ml G418. Homologous recombination in ES cells was first screened by PCR and then confirmed by Southern blotting with several combinations of restriction enzymes and probes shown below.

ROSA26-5' (*EcoR*V-*Sal*I) probe: nucleotides 181,927–182,316 (in AC155722)
ROSA26-3' (*Xba*I-*Xba*I) probe: nucleotides 173,746–174,359 (in AC155722)
3'-neo (*Pst*I-*Bam*HI, 621 bp) probe: 918–1,543 (in U43611; Genbank)

Chimeric mice were generated by a coculture method using eight-cell embryos from CD1 mice (ICR, Charles River Laboratories), bred with CD1 females, and germ line transmission of the mutant allele was determined by PCR and Southern blot analyses.

TgM ubiquitously expressing cre recombinase were mated with knock-in mice to partially or completely execute Cre-loxP recombination, which was confirmed by PCR and Southern blot analyses of tail DNA of offsprings.

[Primers for enhancer allele]

**Table:** 

LCR:	ROSA5FL-5S2: 5'-CCCTCGTGATCTGCAACTCC-3'
	ROSA-LCR2: 5'-TCACTTTTGGAGGTCAGGAA-3'
ε-β:	BT-1S: 5'-AACTGTGTTCACTAGCAACCTCAA-3'
	EP-1A: 5'-GGGCTTGAGGTTGTCCATGTTT-3'
Neo^r^:	Neo-S: 5'-AGAGGCTATTCGGCTATGAC-3'
	Neo-AS: 5'-CACCATGATATTCGGCAAGC-3'

[Primers for promoter allele]

**Table:** 

γ-β:	BT-1S & GM-1A: 5'-CCTTGAGATCATCCAGGTGCTTT-3'
3'HS1:	h3'HS1-5S: 5'-AGAAAGTTTGATGAACTACTTCTGACCC-3'
	h3'HS1-3A: 5'-GACACCCACACATGTCCTGCC-3'
Neo^r^:	Neo-down-5S2: 5'-GACAGAATAAAACGCACGGGT-3'
	ROSA-3A2: 5'-TGGGGCTAAAATGAGTGTTC-3'

[Southern blot probe]

ROSA-3'-383 probe (*Sca*I-*Hin*dIII 390 bp): 179,269–179,658 (in AC155722)

### Animal procedures

Mice were housed in a pathogen-free barrier facility in a 12-hour light/12-hour dark cycle, and fed standard rodent chow. Adult mice were sacrificed by cervical dislocation and the organs were immediately removed and flash-frozen in liquid nitrogen.

Animal experiments were performed in a humane manner under approval from the Institutional Animal Experiment Committee of the University of Tsukuba. Experiments were performed in accordance with the Regulation of Animal Experiments of the University of Tsukuba and the Fundamental Guidelines for Proper Conduct of Animal Experiments and Related Activities in Academic Research Institutions under the jurisdiction of the Ministry of Education, Culture, Sports, Science and Technology of Japan.

### Expression analysis

Total RNA was extracted from phenyl hydrazine-induced anemic adult spleens (1 to 2 months old) or fetal liver (e14.5) by ISOGEN (Nippon Gene) and converted to cDNA using ReverTra Ace qPCR RT Master Mix with gDNA Remover (TOYOBO). One-fortieth of the reaction mixture was subjected to quantitative PCR amplification using the KOD SYBR qPCR Mix (Toyobo) and thermal Cycler Dice (TaKaRa Bio) with the following parameters: 95°C for 5s and 60°C for 30s, 40 cycles. The PCR primer sets used for human β(γ)- or β(ε)-globin genes amplification were GM-1S2 and BT-3A3 (126-bp amplicon) or BT-1S3 and EP-3A (152-bp), respectively. Primer set common to both β(γ)- and β(ε)-globin genes amplification was BT-4S1 and BT-4A1.

GM-1S2: 5'-GCCATAAAGCACCTGGATGAT-3' (39,811–39,831 in HUMHBB)
BT-3A3: 5'-GGCCAGCACACAGACCAGCACG-3' (63,493–63,514)
BT-1S3: 5'-CAACTGTGTTCACTAGCAACCT-3' (62,153–62,174)
EP-3A: 5'-GGGTCCAGGGGTAAACAACG-3' (19,761–19,780)
BT-4S1: 5'-GTGGATCCTGAGAACTTCAG-3' (55,212–55,231)
BT-4A1: 5'-GATAGGCAGCCTGCACTGGT-3' (63,541–63,560)

The primer sets used for mouse endogenous gene expression analyses were as follows:

Thumpd3: 5'-AGTGAGAGAGAAACTGAAGTCGGC-3' and 5'-AAACTCCTGAACAACCACAAACAA-3'.
Setdb5: 5'-GCTAGTCGTTCCAACACTCCTCTG-3' and 5'-AGCCAGGTCAGGATGATTGCAGTT-3'.
βh-globin: 5'-TGGACAACCTCAAGGAGAC -3' and 5'-AGTAGAAAGGACAATCACCAAC-3'.
βmajor-globin: 5'-ATGCCAAAGTGAAGGCCCAT -3' and 5'-CCCAGCACAATCACGATCAT -3'.
α-globin (MAgI-I&II): 5'-TGAGGTCAATGAAGGGGTCGT-3' and 5'-CCTTTCCAGGGCTTCAGCTCCATAT-3'.
GAPDH: 5'-AAAATGGTGAAGGTCGGTGTG-3' and 5'-TGAGGTCAATGAAGGGGTCGT-3'.

### ChIP analysis

The animals (2 to 4 months old) bearing both the *LCR+β(ε)* and *β(γ)+3*'*HS1* knock-in alleles in *trans* were made anemic and nucleated erythroid cells were collected from their spleens. Following fixation with 1% formaldehyde for 10 min at room temperature. Nuclei (2 x 10^7^ cells) were digested with 12.5 units/ml of micrococcal nuclease at 37°C for 20 min. The chromatin was incubated with anti-CTCF antibody (D31H2; Cell Signaling Technology) or purified rabbit IgG (Invitrogen) overnight at 4°C and was precipitated with preblocked Dynabeads protein G magnetic beads (Life Technologies, Carlsbad, CA). Immunoprecipitated materials were then washed and reverse cross-linked. DNA was purified with the QIAquick PCR purification kit (Qiagen, Venlo, The Netherlands) and subjected to qPCR analysis. The endogenous *H19* ICR and *Necdin* sequences were analyzed as positive and negative controls, respectively [41]. LCR-HS5 and 3'HS1 primer sets were as follows:

HS5-CTCF-5S2: 5'-GGTCACAGAATAACCTGAGT-3'
HS5-CTCF-3A: 5'-CAAAAGGGCTCCTTAACAAC-3'
3'HS1-CTCF-5S2: 5'-TCACTGAAGTAGGGAGGGAAGAA-3'
3'HS1-CTCF-3A2: 5'-AAGGTCATTCCTTTAATGGTCTTTTC-3'

## Results

### Generation of enhancer and promoter knock-in alleles at the mouse *Rosa26* locus

A targeting vector for the enhancer allele (Fig 1B, top) carried the LCR (HSs 1~5) and the β-globin gene sequences. The one for the promoter allele (Fig 1C, top) carried the β-globin gene and the 3’HS1 sequences. To distinguish human β-globin gene transcripts expressed from each allele by PCR, portions of the β-globin gene were replaced either with corresponding segments of the ε- and γ-globin genes in the enhancer and promoter targeting vectors, respectively. In this experimental design, expression of both chimeric genes is under the control of a common human β-globin proximal promoter element (1.6 kb).

In the absence of a *cis*-linked LCR, the endogenous human β-globin gene locus becomes heterochromatinized (e.g. in the Hispanic thalassemia patient; [42, 43]). In addition, expression of β-like globin genes without linked LCR in transgenic mice frequently suffer from position of integration site effect [44]. Therefore, a β-globin transgene without an LCR enhancer in *cis* on the promoter allele can be heterochromatinized and/or to not be efficiently activated by the LCR on the enhancer allele in *trans*. We therefore chose the mouse *ROSA26* locus (on chromosome 6) for testing the transvection phenomenon, because this locus has a stable open chromatin structure in virtually all tissues [45].

To test for possible involvement, if any, of co-transcription of the β-globin genes in the enhancer and promoter alleles for transvection analysis, an ε-marked β-globin [β(ε)-globin] gene sequence was surrounded by loxP sites (floxed) in the enhancer allele so that it could be removed by conditional *in vivo* cre-loxP-mediated homologous recombination (Fig 1B). To test for involvement of allelic proximity mediated by CTCF factors in the transvection experiment, the 3’HS1 sequence in the promoter allele was also floxed (Fig 1C). Following homologous recombination with these targeting vectors in R1-ES cells, genomic DNA was prepared and correct recombination events were confirmed by Southern blot analyses using several combinations of restriction enzymes and specific probes (Fig 1D and 1E).

### *In vivo* Cre-loxP recombination to derive daughter sublines

**Fig 2:**
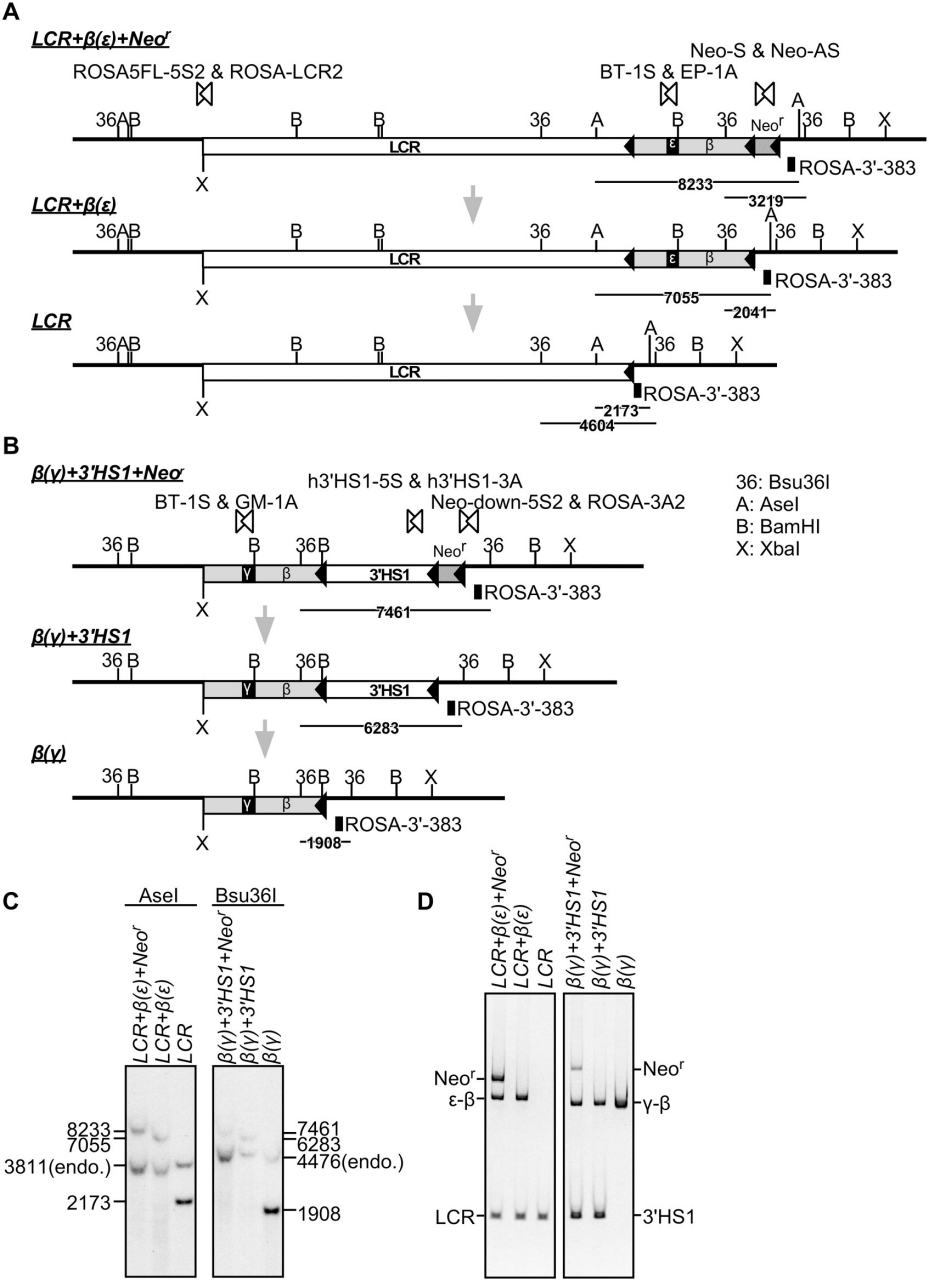
Derivation of enhancer/promoter-allele variants by *in vivo* Cre-loxP recombination. **(A)** Enhancer knock-in mouse bearing the *LCR+β(ε)+Neo*^*r*^ locus was mated with Cre-TgM to induce *in utero*, partial cre-*loxP* recombination, which resulted in selective excision of either Neo^r^ or Neo^r^+β(ε)-globin sequences to generate *LCR+β(ε)* or *LCR* alleles, respectively. A, *Ase*I; B, *Bam*HI; 36, *Bsu*36I. **(B)** Similarly, *β(γ)+3’HS1* or *β(γ)* alleles were derived from the promoter knock-in mouse bearing the *β(γ)+3'HS1+Neo*^*r*^ locus by deletion of the Neo^r^ or Neo^r^+3’HS1 sequences, respectively. **(C)** Successful cre-*loxP* recombination was confirmed by Southern blot analysis. Tail genomic DNA of mutant mice was digested with *Ase*I (enhancer knock-in series) or *Bsu*36I (promoter knock-in series), separated on agarose gels, and Southern blots were hybridized to the ROSA-3’-383 probe shown in panels A and B. **(D)** Each allele was discriminated by multiplex PCR analyses of tail genomic DNA from mutant mice. The LCR, ε-βand Neo^r^ sequences in the enhancer knock-in alleles were amplified by PCR primers shown by paired open arrowheads in panel A. The γ-β, 3’HS1 and Neo^r^ sequences in the promoter knock-in alleles were amplified by PCR primers shown by paired open arrowheads in panel B.

Following establishment of germ line modified mouse lines from the mutant ES cells by co-culture aggregation, the one carrying the enhancer knock-in allele (*LCR+β(ε)+Neo*^*r*^; Fig 2A, top) was mated with *cre*-expressing TgM to remove either the “Neo^r^” or “Neo^r^+β(ε)-globin gene” sequences, thereby generating either “*LCR+β(*ε)” or “*LCR*” alleles, respectively (Fig 2A). Similarly, “*β(γ)+3’HS1*” and “*β(γ)*” alleles were derived from lines carrying the promoter knock-in allele (*β(γ)+3’HS1+Neo*^*r*^) by removing “Neo^r^” or “Neo^r^ +3’HS1” sequences, respectively (Fig 2B). Correct cre-loxP recombination events were confirmed by Southern blotting (Fig 2C), as well as PCR analyses of tail genomic DNAs (Fig 2D).

### Evaluation of enhancer activity *in vivo*

**Fig 3:**
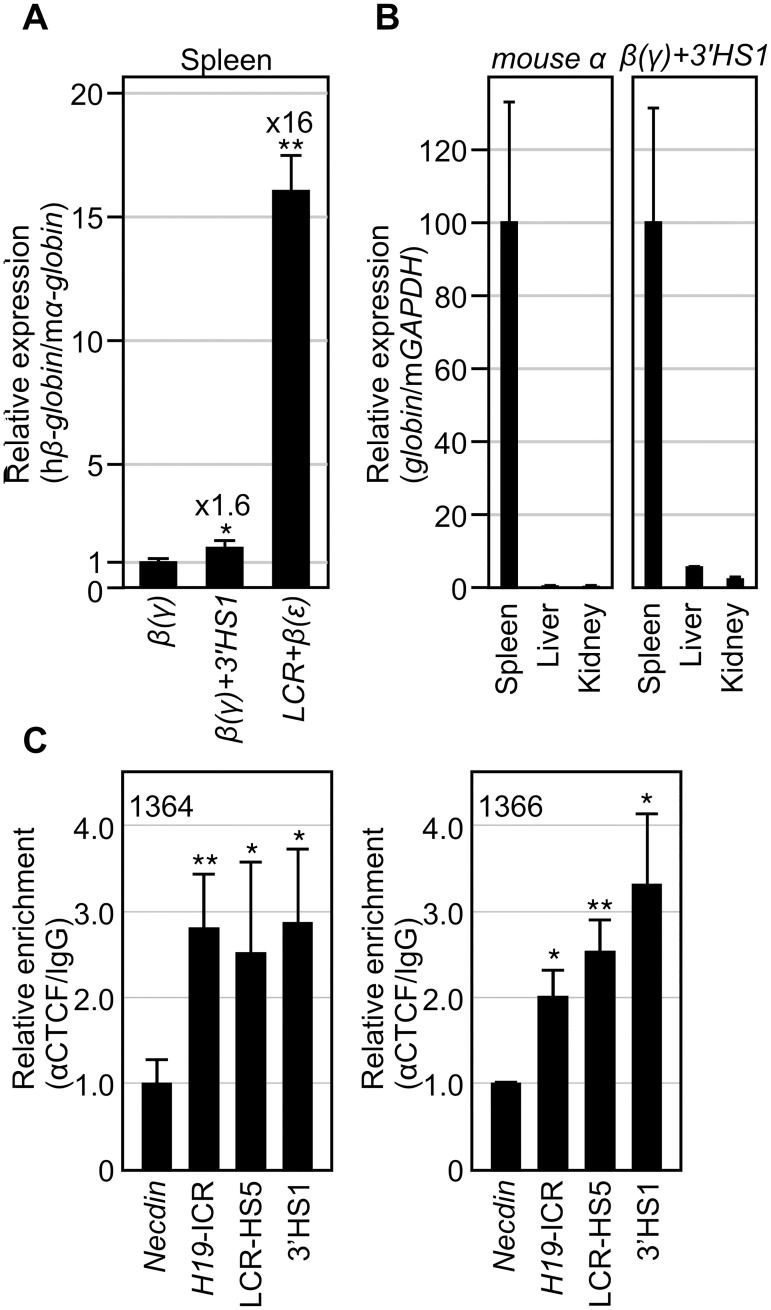
Expression of human β-globin genes in knock-in mice. **(A)** Total RNA was prepared from spleens of 1-month-old anemic mice (N = 4 for each genotype). Expression levels of the human β(γ)- or β(ε)-globin genes (analyzed by common primer set targeted at β-globin sequence; BT-4S1 and BT-4A1) and endogenous mouse α (mα)-globin gene were analyzed by qRT-PCR. The ratio of hβ/mα-globin genes was calculated (the expression value of the β(γ)-globin was set at 1). P values (vs *β(γ)*: *<0.05; **<0.01. **(B)** Total RNA was prepared from spleens, livers and kidneys of 1-month-old anemic mice (N = 4 for each tissues). Expression of mα-globin, β(γ)-globin and endogenous mouse (m)GAPDH genes was analyzed by qRT-PCR. The expression levels of mα- (left panel) or β(γ)-globin (right) genes, both compared to that of mGAPDH gene were calculated (the expression values in spleen samples were set at 100). **(C and D)** ChIP was conducted for CTCF in the spleen cells of anemic animals bearing both *LCR+β(ε)* and *β(γ)+3’HS1* alleles. The *Necdin* gene and the *H19* ICR sequences were analyzed as negative and positive controls, respectively. Quantitative PCR was repeated at least three times for each sample. Fold enrichment of CTCF relative to IgG control (average values with S.D.) was calculated and graphically depicted (average value of negative controls was set at 1.0). P values (vs *Necdin*): *<0.05; **<0.01.

To analyze chimeric β-globin gene expression in the knock-in mutant alleles, adult mice were made anemic, nucleated erythroid cells were collected from their spleens and recovered RNAs were reverse-transcribed. Expression of hybrid β(γ)- and β(ε)-globin genes was analyzed using a primer set comon to both chimeric gene sequences. Addition of 3’HS1 to the β(γ)-globin gene increased its expression level by only 1.6-fold *in vivo* (Fig 3A), which was less prominent when compared with its effect in YAC TgM [46]. In human β-globin YAC TgM, deletion of 3’HS1 sequences attenuated adult β-globin gene expression by more than 10-fold, possibly because this sequence plays additional roles in higher order chromatin organization at the human β-globin locus in the 150-kb YAC TgM [47].

The expression level of the β(ε)-globin gene linked to the LCR in *cis* was 16-fold higher than that of the β(γ)-globin gene in isolation (Fig 3A). This magnitude seemed much less significant than when LCR is deleted from the whole locus in endogenous [43] or transgenic environments [44]. It is generally accepted that the LCR not only potentiates promoter activity as an activator but also opens chromatin [43]. While this latter activity is a part of the “enhancer” function in the context of the native β-globin locus, the *Rosa26* locus is in an open chromatin configuration by its nature and therefore, the observed 16-fold activation may represent a promoter potentiation function of the LCR alone.

### Evaluation of read-through transcription from the *Rosa26* promoter

Because the enhancer and promoter constructs were integrated at the ubiquitously expressed *Rosa26* locus, some portion of the chimeric β-globin gene transcription could be driven by the *ROSA26* gene promoter. We therefore quantified how much read-through transcription from the *Rosa26* gene promoter might contribute to expression of the β-globin gene sequence (Fig 3B). Total RNA was extracted from the spleen, liver and kidney of anemic adult mice and the expression levels of β(γ)-globin+3’HS1 gene and the mouse α-globin gene, relative to the GAPDH gene expression, were determined by qRT-PCR. The mouse α-globin gene was preferentially expressed only in the spleen, as expected (Fig 3B, left). In contrast, while β(γ)-globin+3’HS1 was highly expressed in the spleen, its low level expression was also observed in the liver and kidney. Since α-globin gene expression was barely detected in these non-hematopoietic tissues (in adults), we concluded that contamination of erythroid cells in these tissues was negligible. Therefore, low level β(γ)-globin+3’HS1 gene expression in the liver and kidney (and probably in the spleen) was under the control of the *Rosa26* promoter. In other words, it appears that at least 90% of β(γ)-globin+3’HS1 gene transcription in the spleen initiates from the β-globin gene promoter.

### Confirmation of CTCF binding to the LCR-HS5 and 3’HS1 regions

To test for CTCF binding to well-established CTCF binding sites in LCR-HS5 and 3’HS1 of the human β-globin locus in mutant animals, ChIP analyses were conducted using chromatin prepared from anemic spleen (erythroid) cells (Fig 3C). PCR primers for *H19* ICR and *necdin* [5] loci were included as positive and negative controls for CTCF binding, respectively. As expected, CTCF enrichment was observed at the LCR-HS5 and 3’HS1 regions, confirming its binding to these sites *in vivo*.

### Cross-mating to derive animals carrying distinct pairs of enhancer and promoter alleles

**Fig 4:**
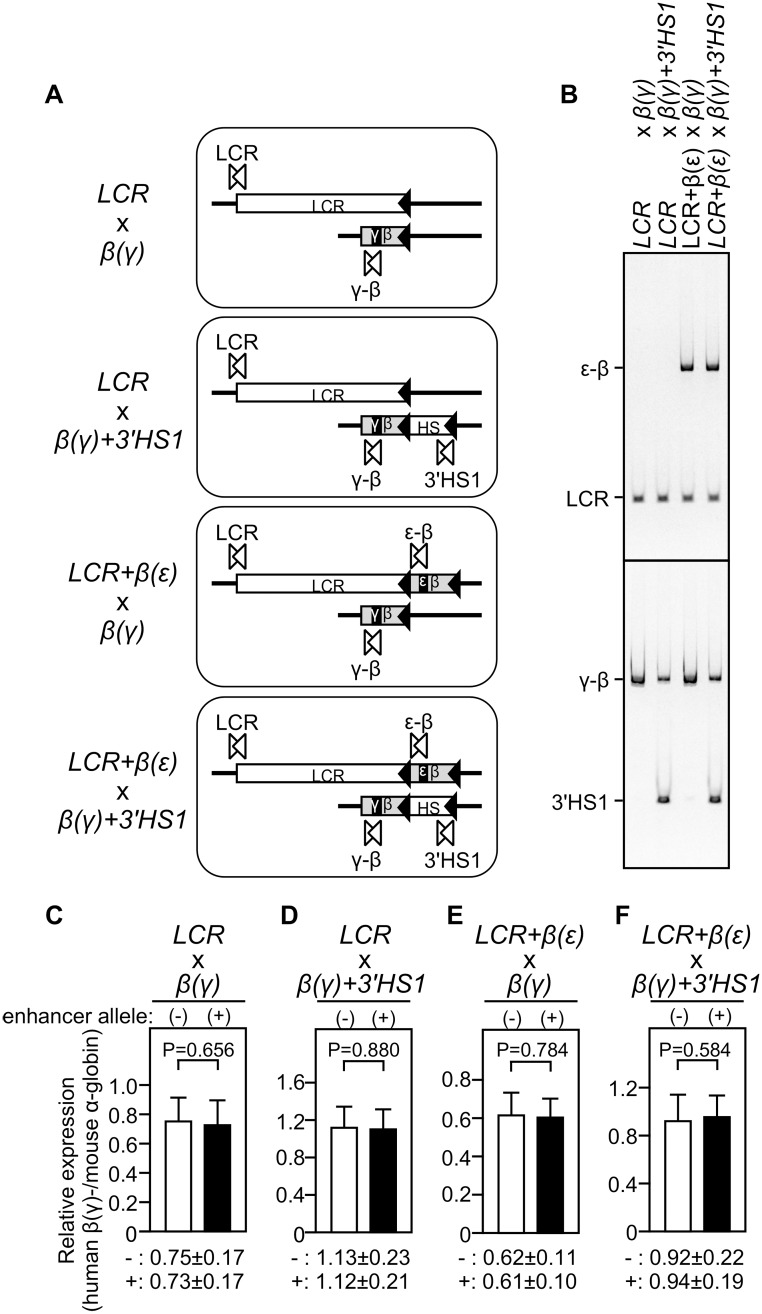
Expression of β(γ)-globin genes in knock-in mice. **(A)** Schematic representation of four different combinations of enhancer and promoter knock-in alleles to test for enhancer-promoter interaction in *trans*. **(B)** Mouse genotypes shown in (A) were confirmed by multiplex PCR analyses of tail genomic DNA of mutant mice. The ε-β, LCR, γ-β and 3’HS1 sequences were amplified by PCR primers shown by paired open arrowheads in panel A. **(C-F)** Total RNA was prepared from spleens of 1-month-old anemic mice. Numbers analyzed for each genotype are shown in the S1 Fig. Expression of β(γ)-globin and endogenous mα-globin genes was analyzed by qRT-PCR. The ratio of hβ(γ)-globin / mα-globin genes was calculated and average value with S.D. was graphically depicted for each genotype group (Although values are arbitrary, they can be quantitatively compared between the panels).

To generate four distinct combinations of enhancer- and promoter-knock-in alleles (Fig 4A), animals carrying heterozygous enhancer alleles (*LCR*^+/-^ or *LCR+β(ε)-globin*^+/-^) and those carrying homozygous promoter alleles (*β(γ)-globin*^+/+^ or *β(γ)-globin+3’HS1*^+/+^) were mated. Theoretically, in the next generation, half of the litters carry both enhancer and promoter alleles in *trans* and the other half carry promoter allele only (Fig 4A). Derivation of animals bearing the expected genotypes was confirmed by Southern blot and allele-specific PCR analyses (Fig 4B).

### The test for transvection-like enhancer-promoter interactions

The expression of the hybrid β(γ)-globin gene was compared in two animal groups carrying either the promoter allele alone or both enhancer and promoter alleles in *trans* (Fig 4C–4F and S1 Fig). Anemic spleens were collected from ~1 month old animals and the expression levels of human β-globin and mouse α-globin genes were determined by qRT-PCR.

Human β(γ)-globin gene expression normalized to that of the mouse α-globin gene in the two groups (with or without the LCR in *trans*) did not differ significantly, providing no evidence for transvection (Fig 4C and S1A Fig). Even when compared within single litters, no significant difference was observed between two groups. This result was consistent with a report by Noordermeer *et al* [38], in which the LCR and γ-globin gene were individually integrated at the *Rad23a* gene locus on mouse chromosome 8 and tested for transvection-like interaction.

Next, the β(γ)-globin gene with an attached 3’HS1 sequence was used as a reporter and the experiment was repeated (Fig 4D and S1B Fig). Because the LCR-HS5 and 3’HS1 sequences were bound by CTCF (Fig 3C), it was possible that the LCR and 3’HS1 come into close proximity, which then facilitates trans-activation of β(γ)-globin gene by the LCR. Even in this idealized experimental setting, however, no significant transvection was observed.

Then, the combination of LCR+β(ε)-globin and β(γ)-globin genes as enhancer and promoter alleles, respectively, was tested (Fig 4E and S1C Fig). Because transcription units in these two alleles share the same transcriptional regulatory sequences (*i*.*e*. the β-globin proximal promoter), it was possible that two alleles would migrate into a shared transcription factory, which would then lead to β(γ)-promoter activation by the LCR enhancer in *trans*, caused by the close proximity of the two alleles. As shown in Fig 4E, however, no sign of transvetion was detected.

Finally, the combination of the LCR+β(ε)-globin and β(γ)-globin+3’HS1 alleles was investigated (Fig 4F and S1D Fig). Although in some of the litters, statistically significant difference in the expression levels in between promoter alone and enhancer+promoter alleles was observed (S1D Fig), this significant but subtle difference dissapeared when the sample number increased.

### Effects of enhancer insertion on endogenous genes expression in *cis* and in *trans*

**Fig 5:**
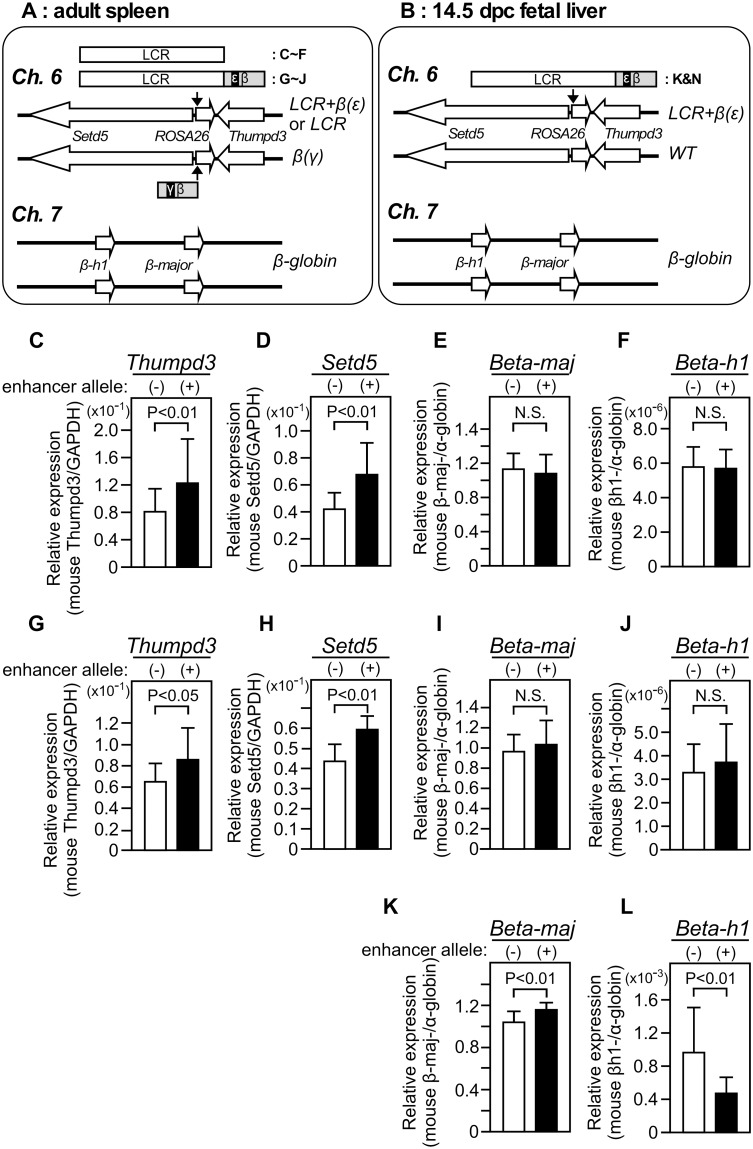
Expression of endogenous genes in knock-in mice. **(A and B)** Schematic representation of combinations of enhancer/promoter knock-in alleles to test for intra- and interchromosomal enhancer-promoter interactions in the adult spleen (A) or fetal liver (B) cells. **(C-J)** The adult spleen was analyzed by qRT-PCR. In addition to the *β(γ)* promoter alleles, animals used in the panels C-F and G-J carried the *LCR* or the *LCR+β(ε)* enhancer alleles (shown in A), respectively. **(K, L)** The fetal liver of the animals bearing the *LCR+β(ε)* enhancer allele (shown in B) was analyzed by qRT-PCR. The ratios of *Thumpd3* and *Setd5* genes expression to that of the *GAPDH* gene (C, D, G and H) or those of *β-major-* and *βh1-globin* genes expression to that of the *α-globin* gene (E, F, I, J, K and L) were calculated and average value with S.D. was graphically depicted. Although values are arbitrary, they can be quantitatively compared between the panels. Sample numbers analyzed in the panels C-F, G-J and K-L are 28, 13 and 16, respectively, in each group.

Because transvection-like activation was not observed in the ectopically inserted test constructs, we next analyzed the effects of enhancer insertion on endogenous gene expression (Fig 5). When enhancer construct (*LCR* alone or *LCR+β(ε)-globin*) was inserted into one of the two *ROSA26* alleles on chromosome 6 (Fig 5A), expression of the surrounding *Thumpd3* (Fig 5C and 5G) and *Setd5* (Fig 5D and 5H) genes was significantly upregulated in the adult spleen. Because these expression values should represent the sum from both alleles, and expression levels of *Thumpd3* and *Setd5* genes in the *β(γ)-globin*-knock-in allele (Fig 5A) are expected to be the same in the presence or absense of the LCR insertion in *trans*, the fold-activation values in the enhancer-knock-in allele alone are predicted to be even higher. Severalfold upregulation of some of the surrounding genes was also observed in the *Rad23a* gene locus after ectopic LCR insertion in *cis* [38]. It is of interst to note that at the *LCR+β(ε)-globin* insertion site (Fig 5A), the endogenous *Thumpd3* and *Setd5* genes (Fig 5G and 5H) as well as the linked *β(ε)-globin* gene (Fig 3A) were activated by the ectopic LCR, data consistent with the flip-flop activation mechanism that was previously proposed [48].

In contrast, interchromosomal *trans*-activation of mouse endogenous βh1-globin (Fig 5E and 5I) and β-major-globin (Fig 5F and 5J) genes on chromosome 7 was not observed in the same adult samples. Because Noordermeer *et al*. reported two-fold *trans*-activation of the endogenous βh1- but not the β-major-globin genes in the fetal liver after ectopic LCR insertion at the *Rad23a* gene locus [38], we next analyzed expression level of these genes at this developmental stage (e14.5 liver). Unexpectedly, while β-major-globin gene expression was slightly upregulated (Fig 5K), that of the βh1-globin gene was even down-regulated (Fig 5L) in the presence of *LCR+β(ε)-globin* insertion in *trans*. The cause of discrepancy between two results may be attributable to position of integration site effect of the LCR insertion.

## Discussion

To gain insight into possible interchromosomal gene regulatory mechanisms, Noordermeer *et al*. employed the human β-globin LCR enhancer and human β-globin promoter as regulatory elements to test for functional consequences (*i*.*e*. gene transcription) of placing them separately at corresponding *cis* locations on homologous chromosomes in mice [38], since these regulatory elements represent one of the most robust and most thoroughly examined enhancer-promoter pairs capable of interacting with each other over extremely long distances [10]. Despite their clear affinity in the native chromatin configuration, they showed no transvection-like interaction when integrated into the *Rad23a* gene locus where many housekeeping genes reside. Our observations at the *ROSA26* locus reported here are consistent with their results (*LCR* x *β(γ)* in Fig 4C).

In contrast, the human β-globin LCR, when integrated at the *Rad23a* gene locus, trans-activated the mouse endogenous βh1-globin gene on chromosome 7 in the fetal liver (e14.5) [38]. It has been reported that coregulated genes (and enhancers) with common temporal and spatial specificities migrate to preassembled PolII factories for transcription and therefore their loci can be in close proximity, even when they are separated by long distances in *cis* on the same chromosome or in *trans* on separate chromosomes [10, 12, 49]. Because both the LCR and β-globin genes are transcribed in erythroid cells [28], and because enhancer-promoter looping in the β-globin gene locus depends on erythroid-specific transcription factors [24, 27, 50], Noordermeer *et al*. proposed that ectopic LCR at the *Rad23a* gene locus modulated endogenous βh1-globin gene transcription through colocalization with a shared transcription factory.

Because the enhancer knock-in allele employed by Noordermeer *et al*. might not be transcribed efficiently because of a missing ERV-9 LTR sequence [29], we introduced the β-globin transcription unit in the enhancer allele at the *ROSA26* locus (*LCR*+*β(ε)* x *β(γ)* or *β(γ)*+3’HS1 in Fig 4A), anticipating its efficient colocalization with the promoter allele in a shared transcription factory. Despite their significant expression in adult erythroid cells (Fig 3A and 3B), however, we did not observe increased reporter gene expression when compared to that in the absence of a paired enhancer allele in *trans* (Fig 4E and 4F). In contrast, insertion of the human β-globin LCR (*LCR*+*β(ε)*) at the *ROSA26* locus exhibited moderate modification of the mouse endogenous β-like-globin gene expression in the fetal liver (e14.5) (Fig 5K and 5L). Our results are thus consistent with those by Noordermeer *et al*. [38] in that both studies demonstrated trans-interaction phenomena at this developmental stage. Nevertheless, downregulation of the βh1-globin gene in the present study contradicts the reproducible two-fold transactivation of the gene in their study [38]. Genome-wide interaction analysis by Schoenfelder *et al*. identified the region around the *Rad23a*, but not the *ROSA26* loci, as a significant interaction site of the endogenous α- or β-globin gene loci [12]. Therefore, integration site-dependent difference in the composition of LCR binding factors might have opposite transcriptional effects on the βh1-globin gene. Another possibility is that addition of the adult-type β(ε)-globin gene cassette to the ectopic LCR at the *ROSA26* locus induced its association with the endogenous β-major-globin gene for co-transcription (Fig 5K), which then caused down-regulation of βh1-globin gene (Fig 5L) as a result of competition for the neighboring, slightly upregulated β-major-globin gene in *cis*.

It has been suggested that chromatin connectivity is not necessarily coupled to transcriptional events [3]. In the β-globin gene locus, for example, erythroid-specific gene activation via active chromatin hub formation turned out to be a multistep process; *i*.*e*. the chromatin loop between the LCR-HS5 and 3’HS1 was preformed in erythroid progenitor cells in a CTCF-dependent fashion prior to globin gene transcription [21]. Importantly, this developmentally early structure was not affected by EKLF ablation in the previously cited knock-out experiment [27]. It can therefore be assumed that, upon erythroid cell maturation, this preformed structure (the outer loop) may facilitate subsequent gene activation that accompanies inner loop formation between an enhancer and promoter, only when cell type-specific transcription factors are present. In addition, higher order pre-structures of the *Shh* and the *HoxD* loci have been proposed to facilitate future promoter–enhancer contacts over very long distances [3, 51]. Therefore, to facilitate enhancer-promoter interactions in *trans*, CTCF binding sites were placed in both enhancer and promoter alleles in our experiment (Fig 4D and 4F). Even under these idealized conditions, we failed to observe upregulation of the β(γ)-globin gene expression. The *cis*-interaction between the LCR-HS5 and 3’HS1 found at the endogenous β-globin locus (outer loop) may not be sufficiently stable to facilitate LCR-β promoter interaction (inner loop) when the two genetic elements are situated in *trans*. Data obtained by the ENCODE Project and available from the UCSC mm9 Genome Browser show that the 200 kb surrounding the *Rosa26* locus contained considerably fewer CTCF binding peaks than the *Rad23a* locus (both in the liver and spleen; S2 Fig). For the formation of *trans*-interchromosomal interactions, both sporadic distribution or specific pairs of CTCF binding sites may be critical to achieve a proper configuration of the locus for activation.

In summary, we reevaluated the possibility of transvection-like interchromosomal gene activation at the *Rosa26* alleles in knock-in mice. Similar to the previous study by Noordermeer *et al*., however, productive transvection-like activation of the β-globin reporter gene by the LCR was not observed even in the presence of a functional CTCF-binding site (3’ HS1) adjacent to the reporter gene. It must be noted that our results shown here do not rule out the possibility of very rare incidence of physical interactions between two homologous mammalian loci in *trans*. Meanwhile, expression of endogenous β-like-globin genes was moderately changed by the ectopic LCR insertion, consistent with the idea that a trans-activation mechanism exists at least in the certain experimental settings.

## Supporting information

S1 Fig Expression of β(γ)-globin genes in the various combination (A~D) of mutant alleles. Accumulation of β(γ)-globin and endogenous mα-globin gene transcripts in the total RNA from spleens of 1-month-old anemic mice was analyzed by semi-quantitative RT-PCR. The expression ratio of hβ(γ)-globin / mα-globin genes was calculated and average values for each individual were graphically depicted. Presence (+; open bars) or absence (-; solid bars) of enhancer alleles in mice is indicated above each panel. Individuals derived from common litters are marked with same IDs (CB~DI).
(PDF)

S2 Fig CTCF binding at around the *Rad23a* and *Rosa26* gene loci. Distribution of genes and CTCF binding peaks at around the *Rad23a* (top) and *Rosa26* (bottom) gene loci in mouse tissues (liver and spleen). A screen shot of the UCSC Genome Browser mm9 Assembly with CTCF peaks relative to two mouse tissues as determined by the ENCODE project is shown. The *Rad23a* and *Rosa26* genes are highlighted in light green.
(PDF)
