# Supplementary material for: Transvection-like interchromosomal interaction is not observed at the transcriptional level when tested in the Rosa26 locus in mouse
Source: PLoS One. 2019 Feb 14;14(2):e0203099. doi: 10.1371/journal.pone.0203099 (PMC6375575; doi:10.1371/journal.pone.0203099)

Supple. Figure 1

A. LCR / β(γ)-globin

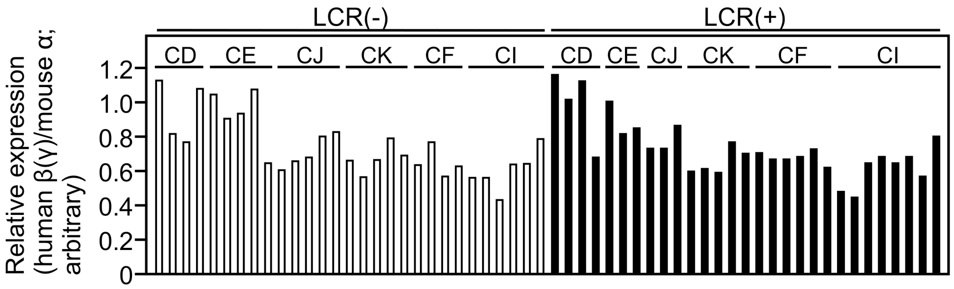

B. LCR / β(γ)-globin+3'HS1

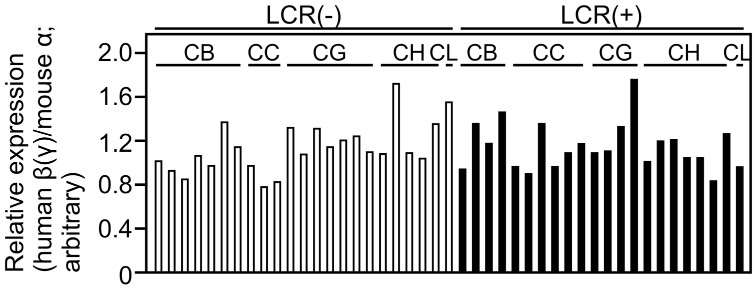

C. LCR+β(ε)-globin / β(γ)-globin

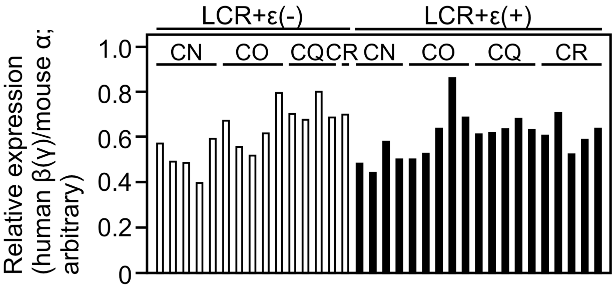

D. LCR+β(ε)-globin / β(γ)-globin+3'HS1

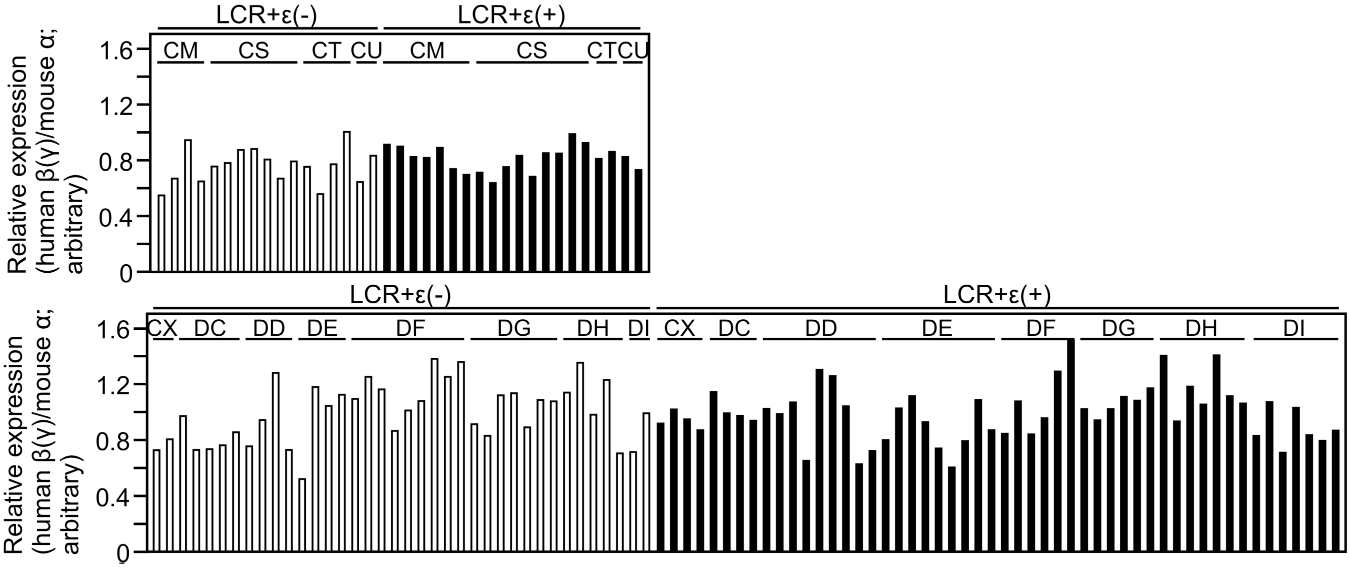

Supplement: S1 Fig — Accumulation of β(γ)-globin and endogenous mα-globin gene transcripts in the total RNA from spleens of 1-month-old anemic mice was analyzed by semi-quantitative RT-PCR. The expression ratio of hβ(γ)-globin / mα-globin genes was calculated and average values for each individual were graphically depicted. Presence (+; open bars) or absence (-; solid bars) of enhancer alleles in mice is indicated above each panel. Individuals derived from common litters are marked with same IDs (CB~DI). (PDF) [file pone.0203099.s001.pdf]
