# Supplementary figures and images for: Transvection-like interchromosomal interaction is not observed at the transcriptional level when tested in the Rosa26 locus in mouse
Source: PLoS One. 2019 Feb 14;14(2):e0203099. doi: 10.1371/journal.pone.0203099 (PMC6375575; doi:10.1371/journal.pone.0203099)

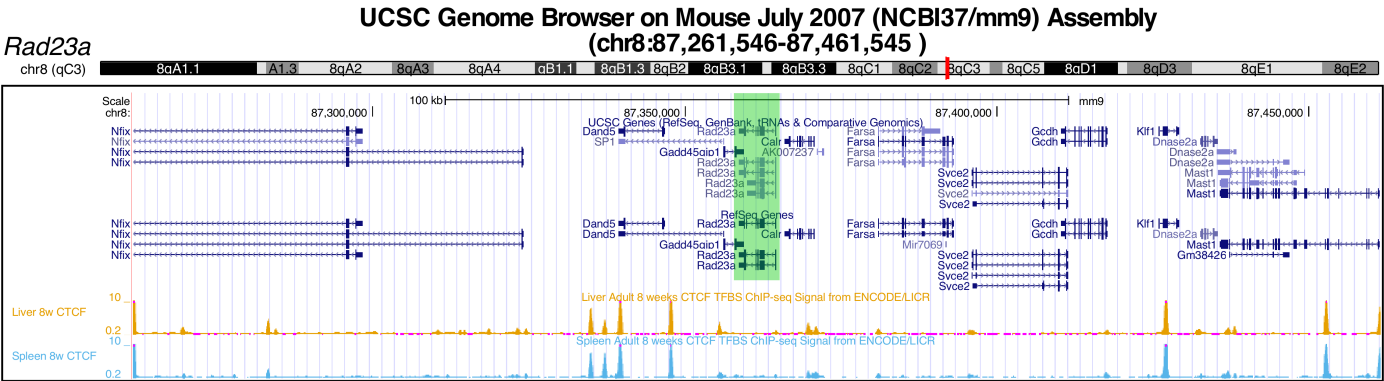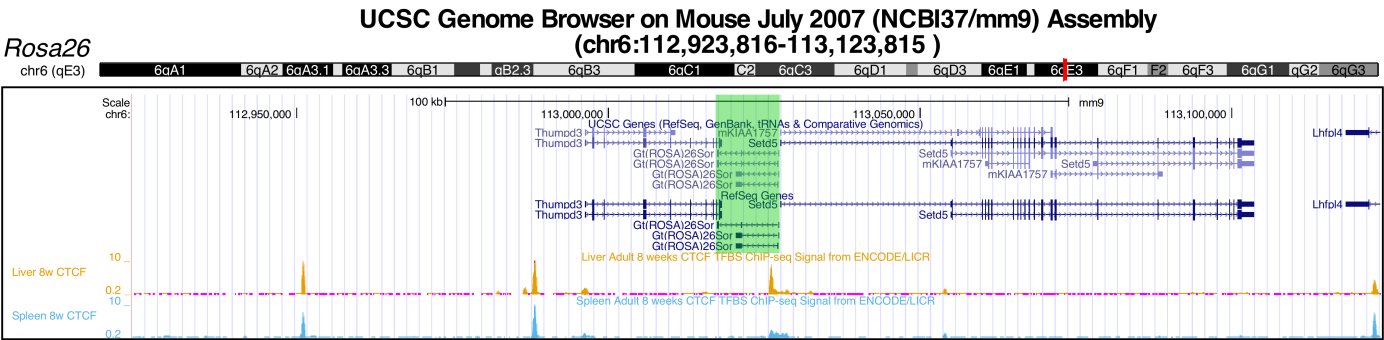

Supplement: S2 Fig — Distribution of genes and CTCF binding peaks at around the Rad23a (top) and Rosa26 (bottom) gene loci in mouse tissues (liver and spleen). A screen shot of the UCSC Genome Browser mm9 Assembly with CTCF peaks relative to two mouse tissues as determined by the ENCODE project is shown. The Rad23a and Rosa26 genes are highlighted in light green. (PDF) [file pone.0203099.s002.pdf]
